# Systematic review and meta-analysis for the impact of rod materials and sizes in the surgical treatment of adult spine deformity

**DOI:** 10.1007/s43390-022-00556-y

**Published:** 2022-07-29

**Authors:** Dawn Bowden, Annalisa Michielli, Michelle Merrill, Steven Will

**Affiliations:** DePuy Synthes Spine, Johnson and Johnson Medical Devices, 325 Paramount Drive, Raynham, MA 02767 USA

**Keywords:** Adult spine deformity, Surgery, Outcomes, Complications, Rods, Meta-analysis

## Abstract

**Purpose:**

To assess clinical and safety outcomes associated with different rod materials and diameters in adult spinal deformity (ASD) surgery.

**Methods:**

A systematic literature review and meta-analysis evaluated ASD surgery using pedicle screw fixation systems with rods of different materials and sizes. Postoperative outcomes (i.e., Cobb, sagittal vertical axis, and pelvic tilt angle) and complications (i.e., pseudarthrosis and rod breakage) were assessed. Random effects models (REMs) pooled data for outcomes reported in ≥ 2 studies.

**Results:**

Among 50 studies evaluating ASD surgery using pedicle screw fixation systems, 17 described rod material/diameter. Postoperative outcomes did not statistically differ between cobalt–chromium (CoCr) vs. titanium (Ti) rods (*n* = 2 studies; mean [95% confidence interval (CI)] sagittal vertical axis angle: CoCr 37.00° [18.58°–55.42°] and Ti 32.58° [24.62°–40.54°]; mean [95% CI] pelvic tilt angle: CoCr 26.20° [22.87°–29.53°] and Ti 20.15° [18.0°–22.31°]). The pooled proportion (95% CI) of pseudarthrosis was 15% (7–22%) for CoCr and 12% (− 8–32%) for stainless steel (SS) (*n* = 2 studies each; Chi^2^ = 0.07, *p* = 0.79). The pooled proportion (95% CI) of broken rods was 12% (1–22%) for Ti (*n* = 3 studies) and 10% (2–19) for CoCr (*n* = 1 study). Among 6.0–6.35 mm rods, the pooled (95% CI) postoperative Cobb angle (*n* = 2) was 12.01° (9.75°–14.28°), sagittal vertical axis angle (*n* = 4) was 35.32° (30.02°–40.62°), and pelvic tilt angle was 21.11° (18.35°–23.86°).

**Conclusions:**

For ASD patients undergoing posterior fixation and fusion, there are no statistically significant differences in postoperative outcomes or complications among rods of varying materials and diameters. Benchmark postsurgical outcomes and complication rates by rod material and diameter are provided.

**Level of Evidence:**

III

## Introduction

Adult spinal deformity (ASD) is a heterogeneous spectrum of abnormalities of the lumbar spine or the thoracolumbar spine that occurs in adult patients [1–3]. Specific ASD diagnoses include primary degenerative sagittal imbalance, iatrogenic spinal deformity, and adult spinal scoliosis [1, 2]. Symptoms of ASD include back and leg pain, numbness, tingling, and weakness [1]. These symptoms can result in functional limitations including difficulty standing upright, bending, and lifting, as well as ambulation and exercise intolerance [1]. The most common causes of spinal deformity in adults are iatrogenic flatback and degenerative scoliosis [1]. The global prevalence of adult spinal deformity is estimated to affect between 32–68% of individuals aged > 65 years, and the numbers of patients with ASD is expected to increase with age progression and a rise in life expectancy [4]. ASD may have a profound impact on a patient’s quality of life; however, there is significant variability in patient presentation [5].

The management of ASD usually begins with medical/interventional treatment with the goals of reducing pain and improving function. Operative treatment may be suggested for patients with progressive deformity, neural compromise, pain, and functional limitations which are not responsive to nonoperative conservative treatment [6, 7]. The aim of operative management of ASD is to restore spinal balance, relieve pain, and achieve solid fusion of vertebral segments. Depending on clinical presentation, a combination of surgical options including decompression, correction of deformity using osteotomies, rod manipulation maneuvers, and fusion may be carried out to achieve these goals [8]. Surgical treatment with pedicle screw fixation systems is a definitive management option for patients diagnosed with ASD.

Recent recognition of the importance of restoring sagittal balance [9], along with advances in surgical techniques and instrumentation have improved postoperative outcomes after ASD surgery; however, there are still opportunities for further improvement [10]. Although surgical management has been found to be beneficial for carefully selected patients, there is a risk of complications including dural tears, deep and superficial wound infections, implant complications, pseudarthrosis, adjacent segment disease, and acute and delayed neurological deficits [10, 11].

Multiple factors contribute to the successful correction of ASD and to minimizing the complications that may arise with surgical treatment [12, 13]. Spinal fixation rods are an important component of pedicle screw fixation systems and may play a significant role in the overall surgical outcomes and in the likelihood of complications [12]. Surgeons require rod options that resist rod fracture and breakage and that deliver the optimal alignment and treatment approach to meet the needs of each patient [12–18]. It is important to gain a better understanding of the rod-specific factors that may contribute to successful surgical and safety outcomes in ASD patients. More specifically, a better understanding of the clinical and economic value of various types of rods available for the surgical management of ASD would help healthcare providers and payers prioritize resource allocation and develop more effective and targeted interventions for the surgical treatment of ASD. Hence, the objectives of this study were to assess current evidence of the postoperative outcomes and complications associated with differing rod materials and dimensions for the operative treatment of ASD. An assessment of the current evidence will identify gaps that will inform priorities for future research.

## Methods

### Study design and approach

The systematic literature review and meta-analysis compared different rod characteristics for the surgical treatment for ASD. The systematic literature review was conducted and reported according to the Preferred Reporting Items for Systematic Reviews and Meta-Analyses (PRISMA) guidelines [19]. The systematic review protocol was registered in the York PROSPERO database (PROSPERO: A registry for systematic review protocols | Augustus C. Long Health Sciences Library [columbia.edu]; PROSPERO 2020).

### Literature search strategy

The literature search was conducted on November 20, 2020 by electronic searching of MEDLINE, Embase, KOSMET: Cosmetic Science, APA PsycInfo, and BIOSIS Previews. The search terms and search strategy utilized were: (spine* OR vertebra*) AND (fusion AND stabilization) AND (rods) AND (adults). The types of studies included were randomized controlled trials (RCTs), non-randomized clinical trials or studies, cohort studies, case control studies, registry studies, economic studies (budget impact and cost-effective analyses), and case series. Relevant secondary research with the highest levels of evidence, specifically systematic literature reviews and meta-analyses, was also included. Study types that were excluded were those that were technical articles, animal/cadaver studies, case reports, editorials, commentaries, and letters. Only English language literature was considered for review. The search was restricted to articles published on or after January 1, 2010.

### Types of participants and interventions

Studies reporting adult patients aged > 18 years at the time of surgery, who had been diagnosed with any kind of spinal deformity (including congenital, degenerative, idiopathic, iatrogenic spinal deformity, flat back syndrome, failed back syndrome) were considered for the study. Adult spine deformity patients with other comorbid conditions were also eligible and were considered for the study. Studies reporting patients aged ≤ 18 years were excluded. Similarly, studies with patients without any spinal deformity were also excluded from the analysis.

Studies reporting any surgical management for spine deformity using any type of rods were included. While the majority of studies included only or mostly posterior pedicle screw fusion and fixation alone, other approaches included anterior lumbar interbody fusion (ALIF), posterior lumbar interbody fusion (PLIF), transforaminal lumbar interbody fusion (TLIF, open or MIS–TLIF) along with the posterior pedicle screw construct. Deformity correction techniques included Ponte osteotomy, Smith–Petersen osteotomy (SPO), pedicle subtraction osteotomy (PSO), and vertebral column resection (VCR). In addition, studies reporting different types of spine surgeries including primary surgeries, secondary surgeries (i.e., patients who already had previous spine surgeries prior to the surgery done during the actual study), or revision surgeries were also included. Studies involving surgical management of spine deformity but not incorporating rods or pedicle screws were excluded. Studies incorporating other conventional non-pharmacological treatments and experimental treatments were also excluded.

### Surgical outcomes

An effort was made to capture and consider major postoperative outcomes and complications in the systematic review and meta-analysis. The postoperative outcomes that were evaluated included postoperative Cobb angle, sagittal vertical axis angle, and pelvic tilt angle. Postoperative complications that were evaluated included pseudarthrosis and rod breakage. Proximal junctional kyphosis (PJK) was not evaluated as there were no studies meeting the inclusion criteria and evaluating specific rod materials and/or diameters that reported PJK.

### Study selection procedure and data extraction

**Fig. 1:**
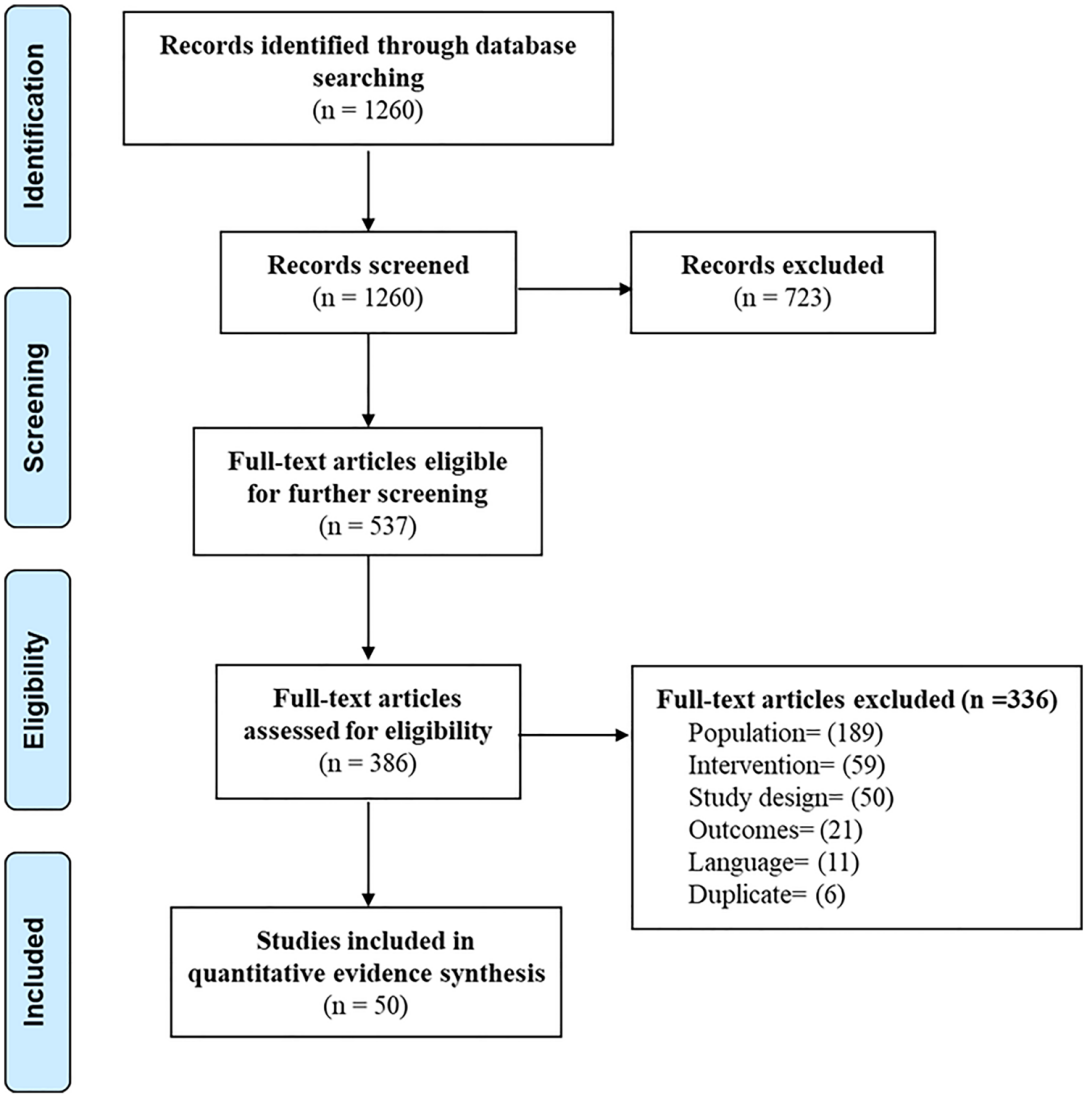
PRISMA flow diagram showing study selection

Two reviewers independently applied the inclusion and exclusion criteria to screen de-duplicated titles and abstracts obtained from the search strategy. Potentially relevant citations were checked in a full-text screening. Disagreements were resolved through discussion and reasons for exclusion were recorded. Figure 1 illustrates the study selection process as a PRISMA flow diagram.

Pre-specified data that were extracted from the relevant studies included the journal citation, study objectives, study design and data source, intervention, comparator, study population (i.e., baseline demographic and clinical characteristics), sample size, duration of follow-up, primary and secondary outcome measures, and author’s conclusions.

### Quality assessment of studies

The principles and methodology of Evidence-Based Practice Guidelines [20] were applied to assess the quality of evidence associated with the performance, safety and cost-effectiveness outcomes from the clinical studies and reports included. Studies were appraised for their level of evidence based on the study design and the rigor of methodology used, as well as the ability to prevent and/or control for biases to analyze cause and effect. All included studies were critically appraised and ranked using the Evidence level and Quality Guide from John Hopkins Nursing Evidence-Based Practice [21, 22].

### Evidence synthesis and statistical analysis

Both qualitative and quantitative synthesis (using meta-analysis) were performed. Qualitative synthesis included summarizing individual studies and describing their results with respect to the relevant outcomes. For the quantitative synthesis, the data were analyzed using RevMan 5.4 and the meta-analysis was performed according to Preferred Reporting Items for Systematic Reviews and Meta-Analyses (PRISMA) guidelines [19]. Pooling and grouping of findings across similar studies and study designs was done. Non-statistical methodology in synthesizing findings across studies of the same level of evidence was applied. Studies that presented and discussed relevant mixed cohort data were analyzed and summarized separately. Meta-analysis was performed for outcomes that were reported in at least two included studies. For continuous outcome measures (length of stay [LOS] and operating room [OR] time), the inverse variance random effects model (REM) was used to estimate the pooled mean difference (MD). The pooled standardized mean difference (SMD) was used for pain scores, since the studies used different pain scales. The mean and standard deviation (SD) were extracted from individual studies or were derived from medians with interquartile ranges or means with *p* values. For dichotomous outcomes, the Mantel–Haenszel REM was used to estimate the pooled risk ratios (RR). For the pooled summary statistics for each outcome in the surgical and non-surgical intervention groups, inverse variance REMs were used. All effect sizes were reported with 95% confidence intervals (CI). The *χ*^2^ test was used to test for statistical heterogeneity (*α* = 0.05) and heterogeneity was quantitatively evaluated using *I*^2^ statistics. Subgroup analyses evaluating the impact of (a) duration of study follow-up; (b) rod material; and (c) rod diameter were also conducted. The statistical significance was set at *p* value ≤ 0.05.

## Results

### Study identification and selection

The literature search yielded 1260 citations which were screened for inclusion. Full texts of 537 of the studies were retrieved for further screening, of which 189 were excluded based on the patient population, 59 based on the intervention, 50 based on the study design, 21 due to lack of relevant outcomes, 11 due to language, and 6 due to duplication of study data. A total of 50 studies evaluating patients aged > 18 years whose surgical management of ASD included pedicle screw and rod systems met the study inclusion criteria and were included in the systematic literature review and meta-analysis (Fig. 1).

### Descriptive characterization of studies

Of the 50 studies evaluating the surgical management of ASD using posterior rods and pedicle screws, 17 studies described the rod material type [23–35]. Fourteen of the 17 studies reported the use of Ti rods either alone [23, 25, 27, 36, 37] or along with other rod materials.[26, 28–32, 35]. Eight studies reported the use of CoCr rods, either alone [24, 26, 28, 36] or along with other rod material [29, 31, 32, 34]. Thirteen studies reported the rod diameter used for the surgical management of ASD [23–35]. The rod diameter varied from 5.0 mm [35] to 6.35 mm [28, 32, 33], and 5.5 mm rods [27, 29]. Table 1 provides a description of the 50 included studies.

**Table 1:** Characteristics of studies (*n* = 50) that fulfilled the inclusion criteria for the systematic review and meta-analysis

Study	Study design	Country	No. of patients	Type of surgery	Type of spine deformity	Gender		Age at surgery (Years)	Follow-up (months)	Outcomes
						Male	Female			
Ohba et al. (2020) [23]	Cohort study	Japan	53	Primary	Combination^a^	27	25	NR	12	ACF
Buell et al. (2020) [24]	Case series	USA	19	Primary, secondary	Degenerative	4	15	67.0 ± 6.5 yr	4.83	ABCDEFGHI
Echt et al. (2020) [38]	Cohort study	USA	106	Primary	Others	NR	NR	68.2 + 9.5	42	ABCE
Marie-Hardy et al. (2020) [36]	Cohort study	France	166	Primary	Degenerative	50	116	67	NR	AB
Prost et al. (2020) [39]	Case series	France	43	Primary	Degenerative	12	31	66.7	NR	BEFI
Daniels et al. (2020) [40]	Case series	USA	40	Primary	Others	9	31	54.2 ± 13.9	26.4 ± 3.6 years	ABCDEF
Sebaaly et al. (2019) [41]	Cohort study	France	290	Primary	Degenerative	63	227	58.4 years	66	BCDE
Lau et al. (2020) [42]	Cohort study	USA	390	Primary	Degenerative	55	235	64.6	NR	ABCDFG
Banno et al. (2019) [25]	Cohort study	Japan	106	Primary	Others	24	82	68	42	ABCDE
Hao et al. (2018) [43]	Case series	China	82	Primary	Others	NR	NR	NR	35	C
Ailon et al. (2018) [44]	Case control study	USA	205	Primary	Degenerative	36	169	56.2 ± 14.8	NR	CDF
Yamato et al. (2018) [45]	Case series	Japan	53	Primary, revision	Combination	10	43	68.5	NR	ABCDF
Gupta et al. (2018) [26]	Case series	USA^b^	49	Primary	Degenerative	22	27	Group 1: 62 years Group 2: 64 years	Group 1: 5 years Group 2: 4 years	ABCDEF
Lewis et al. (2018) [46]	Case series	Canada^b^	66	Primary	Degenerative	21	45	51.7	52.8	BCDEF
Alzakri et al. (2018) [47]	Cohort study	France^b^	10	Revision	Combination	4	6	50.3	48	ABCDEFG
Shen et al. (2018) [27]	Case series	USA^b^	36	Primary, revision	Combination	17	19	69	3.1 years	ACDEFGHI
Banno et al. (2018) [28]	Cohort study	Japan^b^	63	Primary	Others	8	55	68 ± 3.3	42	ABCDE
Levin et al. (2018) [48]	Cohort study	USA^b^	241	NR	Others	112	129	NR	NR	F
Lafage et al. (2017) [49]	Cohort study	USA^b^	252	Primary	Others	NR	0.83	61.5 ± 10.5	24	ABE
Smith et al. (2017) [50]	Registry study	USA	106	Primary	Idiopathic	20	62	60.7 (11.6)	NR	ACDEFGH
Merrill et al. (2017) [29]	Cohort study	USA^b^	31	Primary, revision	Degenerative	9	22	Dual-rod = 68 ± 9; multi-rod = 63 ± 12	36.2 (median)	ABCDEFG
Theologis et al. (2017) [30]	Registry study	USA^b^	183	Primary, secondary	Degenerative	29	154	56 ± 14.8 years	NR	ABG
Park et al. (2017a) [51]	Cohort study	South Korea^b^	40	Primary	Degenerative	8	32	Fish-mouth PSO group: 59.0 ± 15.6; PSO group: 67.6 ± 11.7	NR	ABCDE
Kavadi et al. (2017) [52]	Cohort study	USA^b^	19	Primary, secondary, revision	Combination	NR	NR	NR	30	ACDF
Wang et al. (2017) [53]	Case series	China^b^	35	Primary, secondary, revision	Degenerative	17	18	37.8 ± 12.8	45.5 ± 27.3	ABCDF
Matsumura et al. (2017) [54]	Case series	Japan^b^	15	Primary	Degenerative	0	15	61	46.7	ABCFGH
Park et al. (2017b) [55]	Case series	South Korea^b^	160	Primary, secondary	Degenerative	18	142	67.6 ± 6.1	NR	ABCDEF
Wu et al. (2017) [56]	Case series	China^b^	21	Primary	Degenerative	4	17	58	NR	ABCD
Kim et al. (2018) [57]	Meta-analysis	Australia^b^	973	NR	Combination	NR	NR	NR	NR	B
Wang et al. (2016) [58]	Non-randomized controlled trials	China	16	Primary	Severe coronal/ sagittal deformities	8	8	68.8 ± 9.3	17.7 ± 10.5	ACDEF
Ghogawala et al. (2016) [59]	Randomized controlled trials (RCT)	USA	66	Primary	Degenerative spondylolisthesis	13	53	67	N/R	CDEFGI
Liu et al. (2016) [60]	Case series	China	39	Primary	Late kyphosis deformity	24	15	41	32	ABCDEFHI
Uddin et al. (2015) [61]	Cohort study	USA^b^	71	Primary	Adult degenerative scoliosis	N/R	N/R	MIS Group: 65.68 ± 8.79 open group: 65.68 ± 8.79	MIS Group: 18.16 ± 3.70 open group: 21.82 ± 17.61	BCDEFGHIJ
Soroceanu et al. (2015) [35]	Case series	Canada, USA	246	Primary	ASD	N/R	N/R	N/R	N/R	ABCDEF
Iida et al. (2015) [62]	Cohort study	Japan^b^	814	Primary	Idiopathic	NR	NR	NR	270	ABC
Hyun et al. (2015) [63]	Case serious	USA^b^	56	Primary, Revision	Scoliosis, degenerative sagittal imbalance, ankylosing spondylitis	NR	NR	47.6	8.6	ABCDEF
Omidi-Kashani et al. (2015) [64]	Case serious	Iran	21	Primary	Lumbar spondylolisthesis	14	7	50.4 ± 9.2	39.2	ACDFH
Smith et al. (2014) [31]	Case series	USA	200	Primary	ASD	38	162	54.8 ± 15.8	16.3	ABCDJ
Hyun et al. (2014) [32]	Cohort study	USA^b^	132	Primary	Severe kyphosis and/or scoliosis	NR	NR	NR	NR	ABCDEF
Kim et al. (2014) [33]	Case control study	USA^b^	18	Revision	Pseudarthrosis after PSO	2	16	49.8	104	ABCDEFG
Lewis et al. (2014) [65]	Cohort study	Canada^b^	38	Primary; revision	Thoracic kyphosis	8	30	51.9	NR	ABCDEFGH
Dickson et al. (2014) [66]	Case series	USA^b^	171	Primary, revision	Others	40	131	46.1	NR	ABCEF
Zhu et al. (2014) [67]	Cohort study	China^b^	95	NR	Degenerative	37	58	58.5	93.6	ABCDFG
Akazawa et al. (2013) [37]	Cohort study	Japan^b^	155	NR	Others	32	123	19.0 ± 12.8	46.1 ± 17.8	ABCD
Cho et al. (2013) [68]	Cohort study	USA^b^	67	NR	Others	16	51	64.9 ± 11.1	Failure: 3.5 years non-failure: 3.0 years	BCDF
Scheer et al. (2013) [69]	Cohort study	USA	268	Primary, secondary	Degenerative	38	231	55 ± 15	19.2	ABCEFGI
Kanayama et al. (2013) [70]	Case series	Japan^b^	8	NR	Others	1	7	70	10	ABCDEFG
Crawford et al. (2012) [71]	Cohort study	USA^b^	40	Primary, revision	Others	6	34	30–78 (Range)	NR	ACDEFGH
Smith et al. (2012) [34]	Case series	USA^b^	442	Primary	Others	NR	NR	NR	NR	ACDFH
Martini et al. (2020) [72]	Case series	Germany	77	Revision	Others	11	66	63 (MEAN)	6 and 12	BCDEH

^a^ 53 consecutive patients who underwent minimally invasive lumbar or thoracic spinal stabilization using intraoperative computed tomography image (CT)-guided navigation

^b^ Country based on author’s affiliation

*A* Rod properties, *ASD* Adult spinal deformity, *B* Postoperative outcomes, *C* Patient reported outcomes, *D* Implant failure or malfunction, *E* Complications, *F* Adverse events, *G* Length of stay and surgery, *H* Intraoperative complications, *I* Infections, *J* Cost-data, *PSO* Pedicle subtraction osteotomy, *VCR* Vertebral column resection

### Meta-analyses

#### Impact of rod material

##### Clinical and functional outcomes

**Fig. 2:**
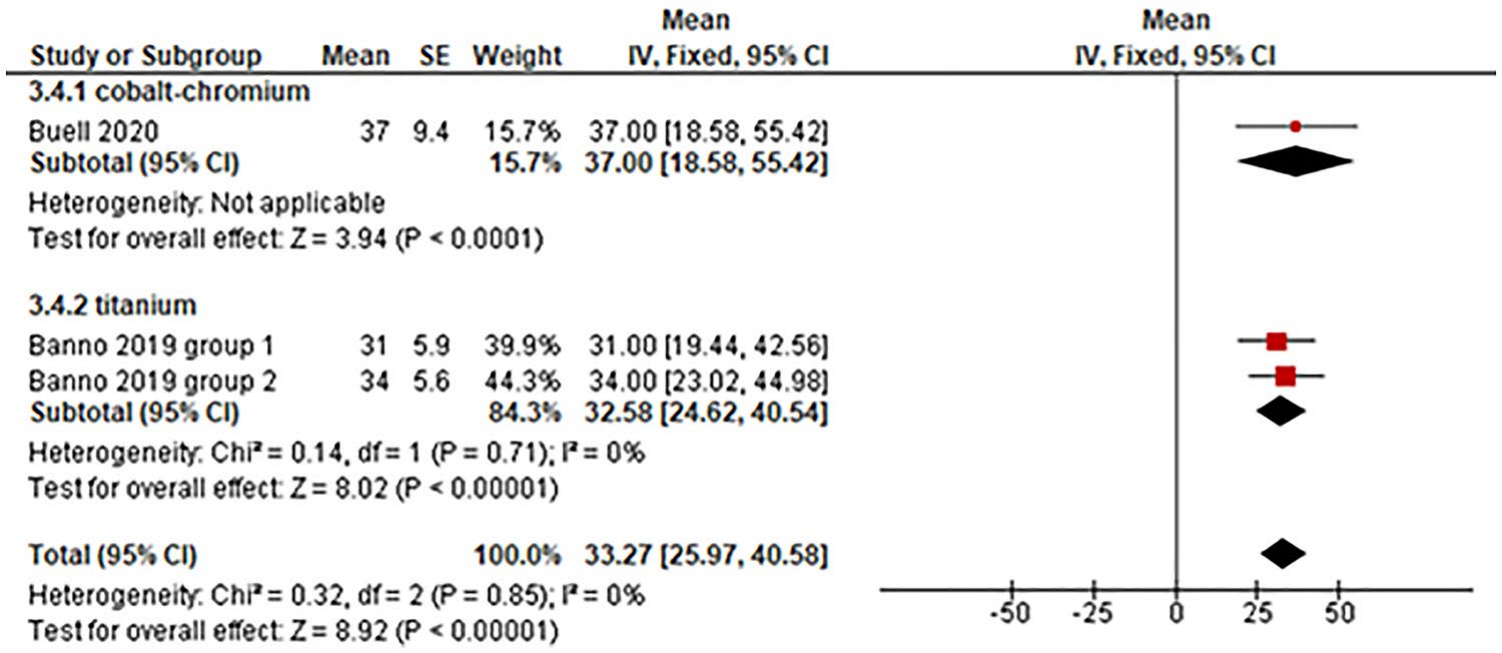
Evaluation of sagittal vertical axis rod material

*Sagittal vertical axis* One study utilized CoCr posterior rods for ASD surgery and reported the postoperative sagittal vertical axis angle of patients (Fig. 2) [24]. The mean postoperative sagittal vertical axis angle with CoCr rods was 37.00° (95% CI: 18.58°–55.42°) [24]. One eligible study with two subgroups that utilized Ti rods reported a mean postoperative sagittal vertical axis of 32.58° (95% CI: 24.62°–40.54°) [25].

**Fig. 3:**
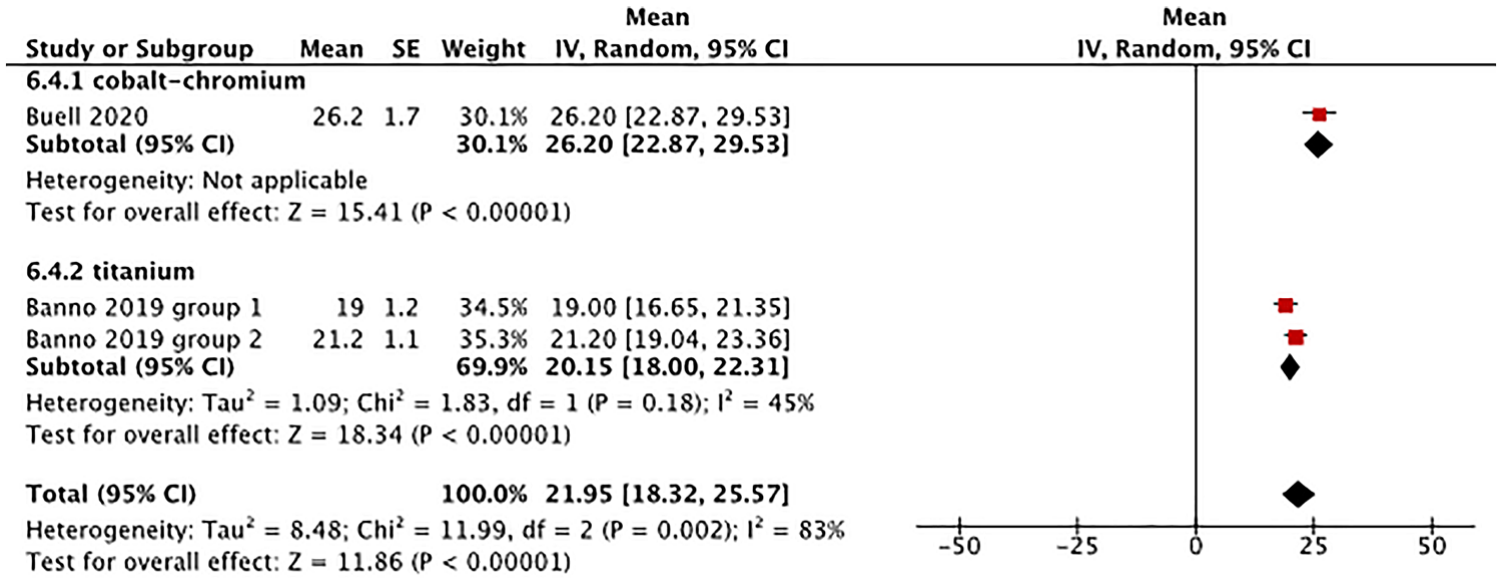
Comparison of pelvic tilt angle by rod material

*Pelvic tilt angle* One study utilized CoCr posterior rods for ASD surgery and reported the postoperative pelvic tilt angle of patients (Fig. 3) [24]. The mean postoperative pelvic tilt angle with CoCr rods was 26.20° (95% CI 22.87°–29.53°) [24]. One eligible study with two subgroups that utilized Ti rods reported the postoperative pelvic tilt angle [25]. The analysis revealed a mean postoperative pelvic tilt angle of 20.15° (95% CI: 18.0°–22.31°).

##### Postoperative complications

**Fig. 4:**
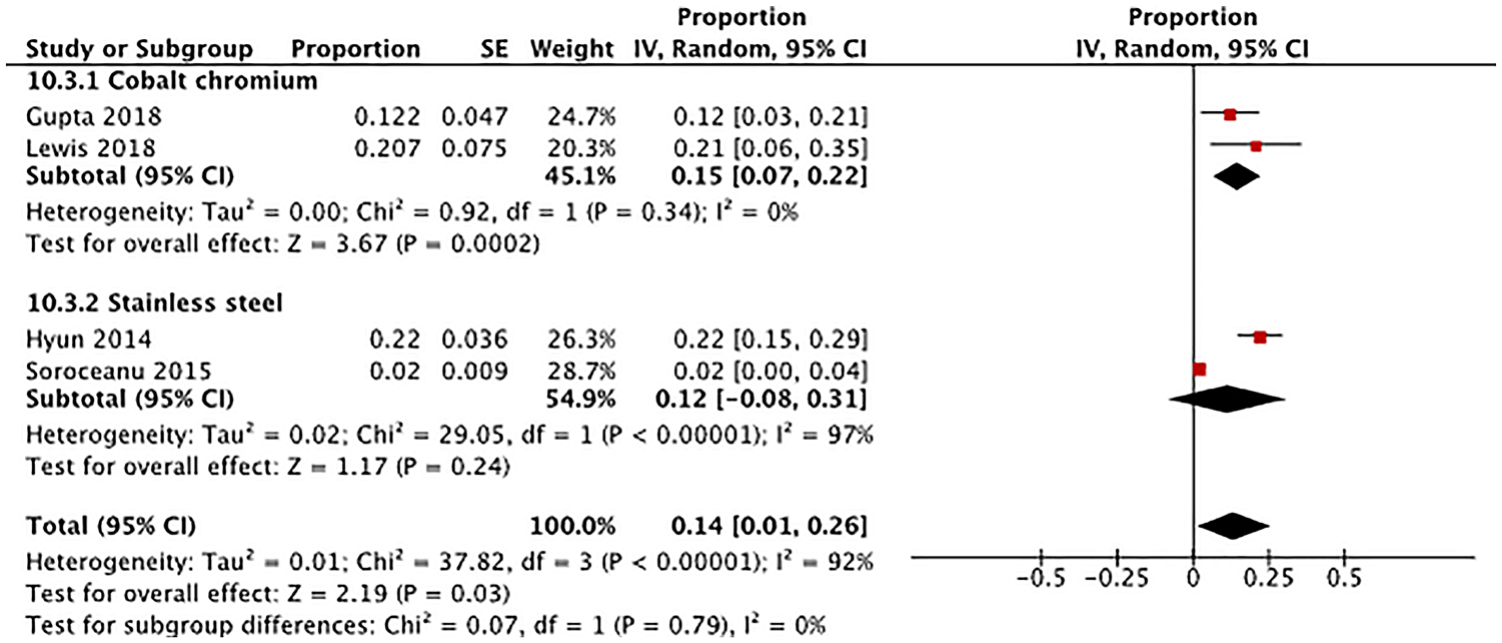
Comparison of pseudarthrosis by rod material

*Pseudarthrosis* Two studies used CoCr rods and reported at least one case of pseudarthrosis in adult patients who underwent spine deformity surgery with pedicle screw fixation systems (Fig. 4) [26, 46]. The overall pooled proportion for pseudarthrosis was 15% (95% CI 7–22%) in patients receiving CoCr rods. Two studies used stainless steel (SS) rods and reported pseudarthrosis [32, 35]; the overall pooled proportion of pseudarthrosis was found to be 12% (95% CI − 8–32%). Test for subgroup differences showed no significant difference in the proportion of pseudarthrosis between the two rod materials (Chi^2^ = 0.07, *p* = 0.79).

**Fig. 5:**
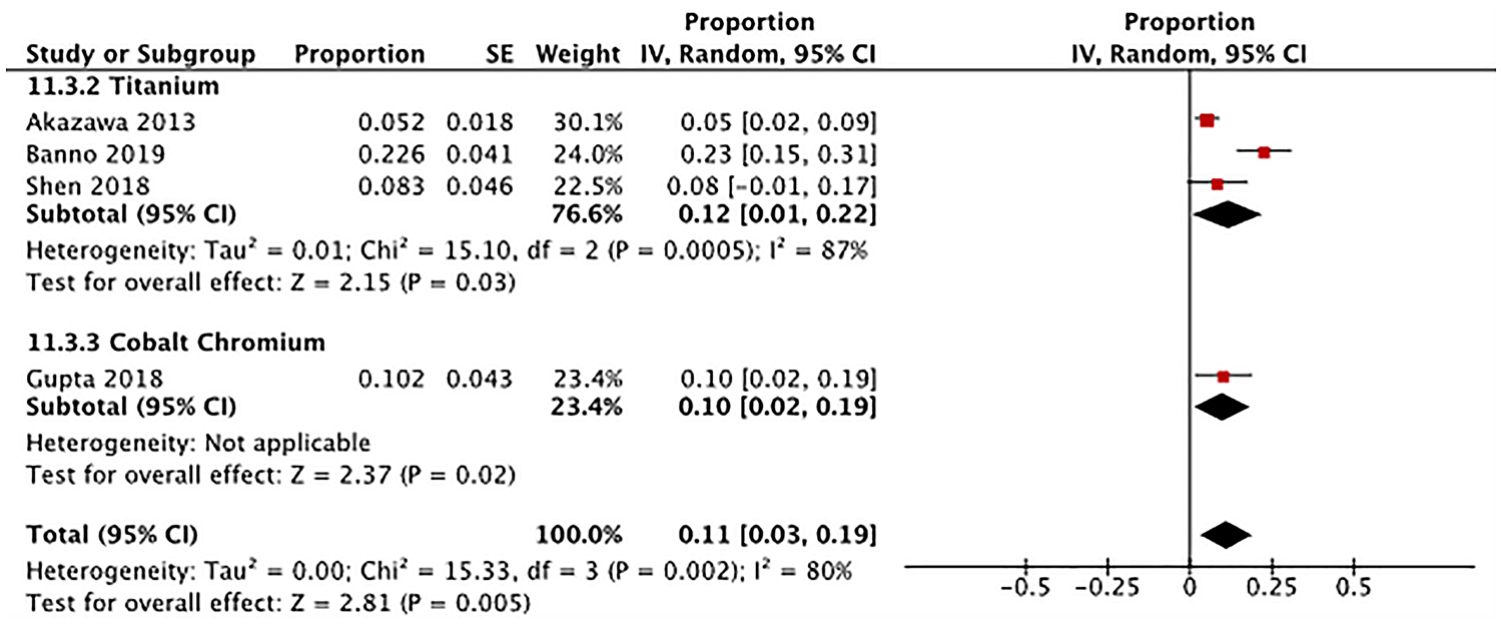
Comparison of rod breakage by rod material

*Rod breakage* Three studies that used Ti rods reported the presence of broken rods in adult patients who underwent spine deformity surgery with pedicle screw fixation systems (Fig. 5) [25, 27, 37]. The pooled subgroup proportion of broken rods was 12% (95% CI 1–22%) in patients that received Ti rods. Only one included study that used CoCr rods reported broken rods in adult patients after spine deformity surgery; the proportion of broken rods was 10% (95% CI 2–19%) [26]. Testing for subgroup differences was not done due to the small number of studies.

#### Impact of rod diameter

##### Clinical and functional outcomes

**Fig. 6:**
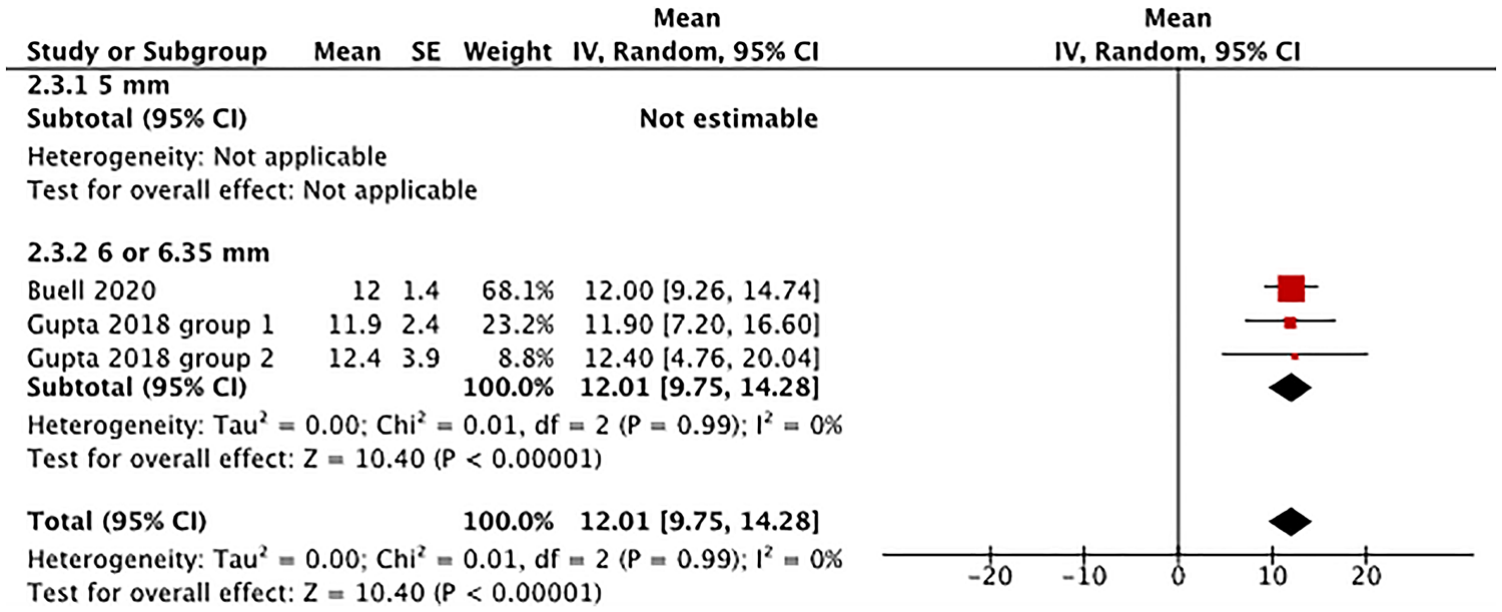
Evaluation of cobb angle by rod diameter

*Cobb angle* Two eligible studies utilized 6.0–6.35 mm posterior rods for ASD surgery and reported data on the postoperative Cobb angle (Fig. 6) [24, 26]. The overall pooled postoperative Cobb angle with 6.0–6.35 mm rods was 12.01° (95% CI 9.75°–14.28°).

**Fig. 7:**
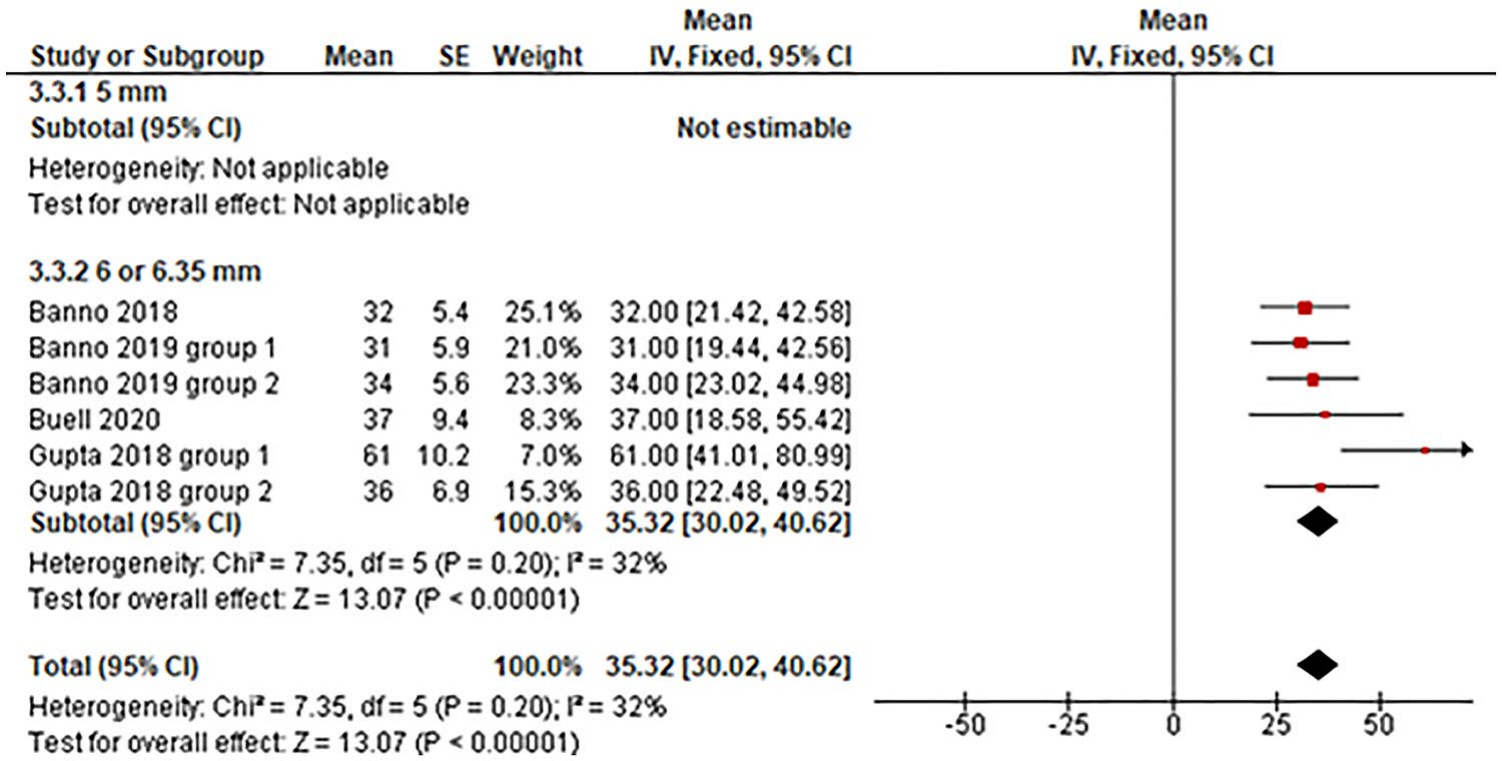
Evaluation of sagittal vertical axis by rod diameter

*Sagittal vertical axis* Four studies utilized 6.0–6.35 mm posterior rods for ASD surgery and reported data on the postoperative sagittal vertical axis angle of patients (Fig. 7) [24–26, 28]. The pooled mean postoperative sagittal vertical axis angle with 6.0–6.35 mm rods was 35.32° (95% CI 30.02°–40.62°).

**Fig. 8:**
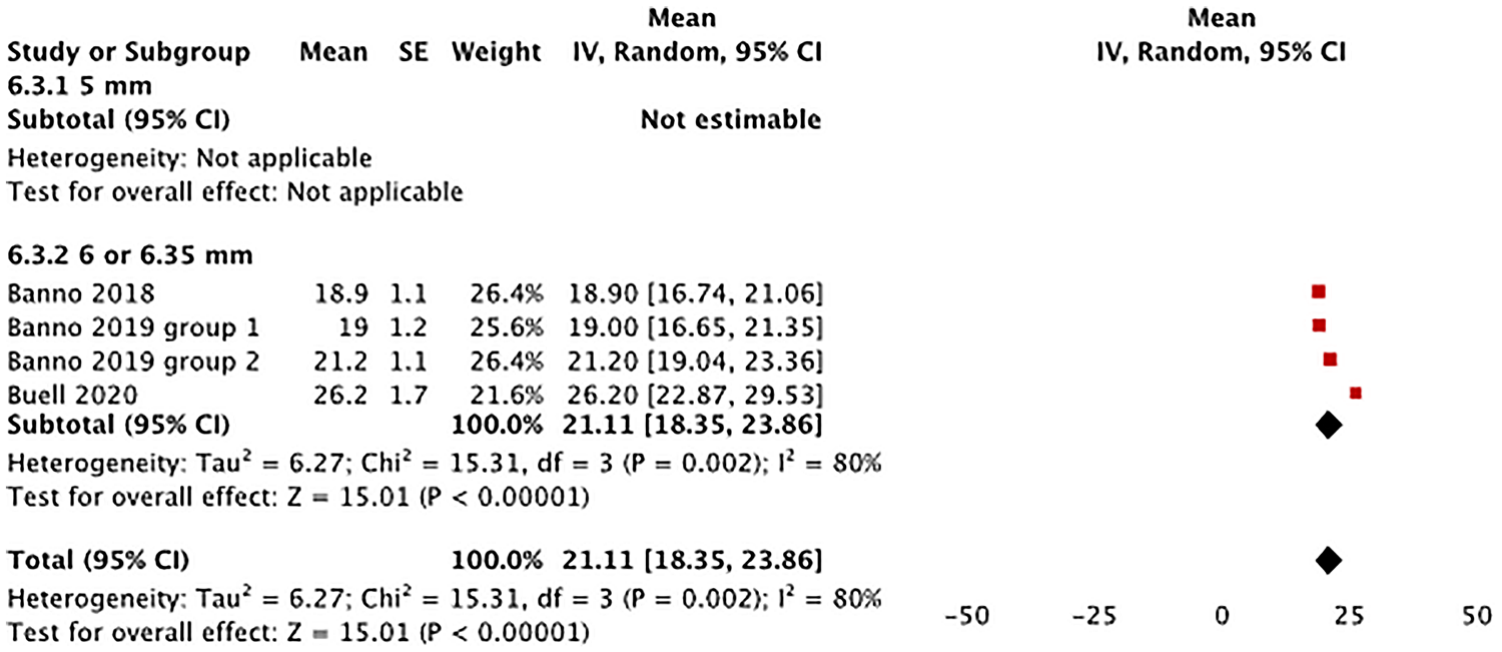
Evaluation of pelvic tilt angle by rod diameter

*Pelvic tilt angle* Three studies utilized 6.0–6.35 mm posterior rods for ASD surgery and reported data on the postoperative pelvic tilt angle of patients (Fig. 8) [24, 25, 28]. The pooled mean postoperative pelvic tilt angle with 6.0–6.35 mm rods was 21.11° (95% CI 18.35°–23.86°). There was a high degree of heterogeneity among included studies (*I*^*2*^ = 80%, *p* = 0.002).

##### Postoperative complications

No studies reported postoperative complication rates by rod diameter.

## Discussion

The choice of rod used for the correction of deformity is an important consideration in the treatment of ASD. The composition and design of the spinal rod must strike a complex balance: the rod must be flexible enough for the surgeon to bend in the desired curve and have a high enough fatigue strength that it does not fracture or break during the therapeutic lifetime of the implant. The ability to resist damage brought about by contouring will depend on the material used and the diameter and shape of the rod. There have been significant changes in the types of rods and the materials used for rods over the years. Initially, Harrington rods consisted of SS. Present-day rod constructs are more likely to consist of either Ti or CoCr. These materials differ in yield strength and stiffness, with Ti having a lower yield strength and lower stiffness, and CoCr having higher yield strength and higher stiffness.

This systematic review and meta-analysis identified 50 qualifying studies evaluating the surgical management of ASD using pedicle screw fixation systems; among which 17 studies described the rod material and rod diameter used. Study findings showed that there was no evidence that surgical outcomes differed by rod material. Two studies reported sagittal vertical axis angle and pelvic tilt angle by rod material and did not find statistically significant differences between CoCr vs. Ti rods (mean postoperative sagittal vertical axis angle: CoCr rods 37.00° [95% CI 18.58°–55.42°] and Ti 32.58° [95% CI 24.62°–40.54°]; mean postoperative pelvic tilt angle: CoCr rods 26.20° [95% CI: 22.87°–29.53°] and Ti 20.15° [95% CI 18.0°–22.31°]). There was an absence of evidence evaluating the impact of rod diameter on postoperative outcomes and complications. Among 6.0–6.35 mm rods, the pooled postoperative Cobb angle was 12.01° (95% CI 9.75°–14.28°), the pooled mean postoperative sagittal vertical axis angle was 35.32° (95% CI 30.02°–40.62°), and the pooled mean postoperative pelvic tilt angle was 21.11° (95% CI 18.35°–23.86°).

In regard to complications, the overall pooled proportion of pseudarthrosis with CoCr rods (*n* = 2 studies) was 15% (95% CI 7.0–22.0%) and with SS rods (*n* = 2 studies) was 12% [95% CI − 8%–32%) (no significant difference; Chi^2^ = 0.07, *p* = 0.79). The pooled proportion of rod breakage with Ti rods (*n* = 3 studies) was 12% (95% CI 1.0% –22.0%) and CoCr rods (*n* = 1 study) was 10% (95% CI 2–19%). No studies reported postoperative complication rates by rod diameter.

Pseudarthrosis is one of the most common complications of ASD surgery, and also one of the most common and costly indications for revision surgery [73, 74]. Studies have also increasingly shown a link between pseudarthrosis and rod fracture [10, 34, 75–77]. Pseudarthrosis has been found to occur in over half of patients with rod fracture and three-quarters of patients with clinically significant rod fracture [12]. This may be explained by the effect of cyclic loading at a non-fused segment, allowing micro-movements to increase construct strain and risk of instrumentation failure [12]. Patients with radiographic evidence of pseudarthrosis after one year postoperatively may have increased risk of rod fracture and may require more careful observation [12].

Rod fracture is a common, problematic complication of ASD surgery, often requiring reoperation [12]. Similar to our current analysis, another recent meta-analysis found that the overall incidence of rod fracture was 12% [13]. The other meta-analysis did not evaluate rod characteristics associated with rod fracture; however, patient factors found to be associated with rod fracture included advanced age, higher body mass index, previous spine surgery, pedicle subtraction osteotomy, a larger preoperative pelvic tilt, and a larger preoperative thoracic kyphosis [13]. Efforts to reduce the incidence of rod fracture have been made, including the use of CoCr rods and multi-rod constructs; [12–18]; however, rod fracture continues to be a significant concern with the currently available rod treatment options and constructs [13]. Hence, there is a need for rods with improved fatigue performance so that breakage and, potentially pseudarthrosis, may be minimized.

The systematic review was designed to cover patients with ASD as comprehensively as possible given the published literature. It identified and summarized 50 studies evaluating the surgical management of ASD in which posterior fixation and fusion was part of the treatment plan. The study delineated the paucity of data available, and it is unfortunate how few of the studies directly compared rod materials and/or diameters. PJK was not evaluated in the current analyses as there were no studies meeting the inclusion criteria and evaluating specific rod materials and/or diameters that reported PJK.

A significant limitation of the meta-analysis component of this study is the heterogeneity of the patient populations evaluated, the variability in the surgical techniques and technologies employed, and the definitions of outcomes used in the analyses [78]. Reasons for such heterogeneity include variability in the definitions of ASD used across the available studies, resulting in varying pathologies and patient populations. The inherent complexity of patient needs and comorbidities, along with patient and surgeon treatment choices based on these complexities, further contributed to the variability. The requirements for customized surgical plans and the availability of published data with results for a specific population with a specific surgical technique hinder the accumulation of sufficient numbers of homogeneous cases for meta-analyses. Hence, we did not restrict our review to particular surgical treatments such as pedicle subtraction osteotomies (PSOs) or vertebrectomies or to types of technologies such as the use of interbody devices or specific grafting material. High volume, multi-center studies with shared definitions and consistent methods of documenting variability will be needed to address the knowledge gaps.

Meta-analysis may offer a way to highlight findings within such heterogeneity, including exposing areas for future research. It also provides a tool for helping to understand the extent of variability [79–81]. In the field of spinal procedures, a growing opinion suggests that inclusion of observational studies in meta-analyses might lead to more robust conclusions without compromising the quality of the results [82, 83]. The current study was conducted in line with recommendations available in the literature for the use of real-world evidence in meta-analyses [84]. Statistical heterogeneity was evaluated using Cochran’s Q test (χ^2^ test) and the I^2^ statistic. Since Q was significant and *I*^2^ was > 50%, it was appropriate to use the random-effects model (REM) to calculate pooled summary estimates. The range of *I*^2^ values observed in the current study (0% to 98%) is not inconsistent with the range of those observed in other meta-analyses of observational data. The heterogeneity present suggests that the meta-analysis covered a broad spectrum of patients with ASD, and the findings establish a foundation for future prospective and retrospective research.

## Conclusions

For patients with ASD, there is a paucity of data evaluating the impact of rod material and rod diameter on ASD postoperative outcomes and complications. However, the current study provides benchmark measures of outcomes and complications for rods of varying material and diameter. Studies that presented postoperative outcomes and complications of ASD surgery by rod material and/or diameter had sizable complication rates. Technologies with improved fatigue performance (i.e., resisting rod fracture or breakage) could improve clinical and functional outcomes and complications.
